# Antioxidative Activity Evaluation of High Purity and Micronized Tartary Buckwheat Flavonoids Prepared by Antisolvent Recrystallization

**DOI:** 10.3390/foods11091346

**Published:** 2022-05-05

**Authors:** Yanjie Liu, Xiaoyu Sui, Xiuhua Zhao, Siying Wang, Qilei Yang

**Affiliations:** 1 College of Chemistry, Chemical Engineering and Resource Utilization, Northeast Forestry University, Harbin 150040, China; klp15lyj@nefu.edu.cn (Y.L.); wsy0822@nefu.edu.cn (S.W.); yql@nefu.edu.cn (Q.Y.); 2 Key Laboratory of Forest Plant Ecology, Ministry of Education, Northeast Forestry University, Harbin 150040, China; 3 Heilongjiang Provincial Key Laboratory of Ecological Utilization of Forestry-Based Active Substances, Northeast Forestry University, Harbin 150040, China; 4 College of Pharmacy, Qiqihar Medical University, Qiqihar 161006, China

**Keywords:** rutin, kaempferol-3-O-rutinoside, quercetin, purification, in vitro human digestion, antioxidant activity

## Abstract

Tartary buckwheat, a healthy food, is associated with a reduced risk of certain human chronic diseases. However, the bioactive component flavonoids in Tartary buckwheat have poor solubility and low absorption in vivo. To improve these points, 60.00% Tartary buckwheat total flavonoids (TFs) were obtained by ethanol refluxing method, which were purified and micronized by antisolvent recrystallization (ASR) using methanol as a solvent and deionized water as an antisolvent. By using High Performance Liquid Chromatography (HPLC) and electrospray ionized mass spectrometry (ESI-MS), the main flavonoid in pure flavonoids (PF) were rutin (RU), kaempferol-3-O-rutinoside (KA) and quercetin (QU); the content of TF is 99.81% after purification. It is more worthy of our attention that micronized flavonoids contribute more to antioxidant activity because of good solubility. These results provide a theoretical reference for the micronization of other flavonoids.

## 1. Introduction

A large number of researchers found that reactive oxygen species (ROS)-induced biochemical changes are major factors in various chronic human diseases, such as diabetes mellitus, cancer, atherosclerosis, and inflammation [1,2,3,4,5,6]. However, flavonoids are abundant in plants, donating hydrogen atom or an electron, chelating metal catalysts, activating antioxidant enzymes, and scavenging oxygen–nitrogen-derived free radicals to effectively inhibit oxidization [7,8]. Since flavonoids are highly effective in oxidation resistance and safer than synthetic antioxidants, they are becoming increasingly popular [9,10].

**Figure 1:**
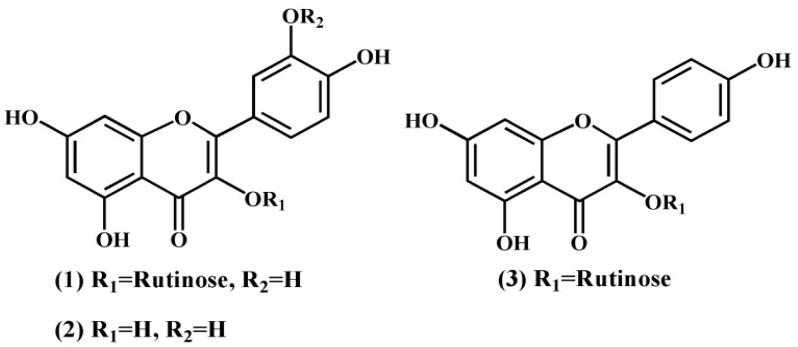
Chemical structures of Rutin (1), quercetin (2), and kaempferol-3-rutinoside (3).

Tartary buckwheat (*Fagopyrum tataricum* Gaertn) is mainly from Asian countries, such as southwest China, northern India, Bhutan, and Nepal [11,12], which have been well explored owing to its long tradition of both medicinal and food crop. Tartary buckwheat seeds (TBS) are rich in flavonoids, which antioxidant, antitumor, hypoglycemic, and hypolipidemic effects [13,14,15,16,17]. Rutin (RU), kaempferol-3-O-rutinoside (KA), and quercetin (QU) are the main flavonoids in TBS, which comprise over 80% of the total flavonoid content [18,19,20]. Their chemical structures are illustrated in Figure 1. Some methods purifying flavonoids from Tartary buckwheat have been introduced. Guo et al. used 85% ethanol solution to recrystallize 52.90% Tartary buckwheat flavonoids, and the content of TF increased to 72.32% [21]. RU, QU, and KA were isolated by high-speed counter-current chromatography in the report of Jiang and coworkers [22]. The purities of these compounds were all above 96.0%. You et al. [23] purified the flavonoid samples from TBS extraction by AB-8 macroporous resin. The flavonoid content increased from 78.42% to 98.73%. However, no study about the purification of Tartary buckwheat flavonoids by antisolvent recrystallization (ASR) is available to date.

Numerous studies showed that the ASR method was applied to reduce the particle size of low-solubility drugs by controlling crystallization speed or obtaining a polymorphic crystal form of drugs with different physicochemical properties by changing crystallization style. Micro- or nanoparticles of drugs can have better solubility and bioavailability due to increasing surface area contact with the dissolution medium [24,25,26,27]. Based on the same principle, the ASR method also can be applied to the separation and purification of the target substance. Zu et al. [28] purified *ginkgo biloba* extract (GBE) by using theASR method, in which the contents of flavonoids and terpene lactones were increased to 53.85% and 11.40%, respectively. Therefore, we aimed to purify crude flavonoids (CFs) from TBS by using the ASR method, to simultaneously obtain purified flavonoid (PF) particles, and explore the effect of micronized flavonoids on digestibility and antioxidant activity.

As a result, this research introduces the ASR technique for purifying crude flavonoid (CF) from Tartary buckwheat seeds. We expect that by reducing particle size, this technique not only enhances the purity of CF but also preserves the key flavonoids and adds to enhanced absorption and antioxidant activity.

## 2. Materials and Methods

### 2.1. Materials

Tartary buckwheat seeds were obtained from Sichuan Jing Cheng Seeds Development Co., Ltd. (Sichuan, China). Reference standards of RU (purity ≥ 99.9%), QU (purity > 98.0%), and KA (purity > 98.0%) were from Shanghai Pharmaceutical Technology Co., Ltd. (Shanghai, China). Ethanol, methanol, formic acid, acetonitrile, and other chemical reagents were supplied from J&K Scientific (Beijing, China). The *Escherichia coli* (ATCC 25922) and *Lactobacillus casei* (ATCC 393) were obtained from Biobw Biotechnology Co., Ltd., (Beijing, China). Deionized water was prepared with Hitech-K flow water purification system (Hitech Instruments Co., Ltd., Shanghai, China). 1,1-diphenyl-2-picrylhydrazyl (DPPH) Malondialdehyde (MDA) test kit was purchased from the Nanjing Jiancheng Bioengineering Institute (Nanjing, China).

### 2.2. Preparation of CF

CF was obtained in advance using an ethanol refluxing extraction (refer to Guo et al. [21]) with slight modifications. A certain amount of TBS has been triturated, and 75% ethanol solutions (*w*/*v*, 1:10) were placed in a round flask. Then, an ethanol reflux extraction was utilized to extract CF in a water bath at 60.0 °C. After 3 h, the composite liquid was divided by centrifugation, and the ethanol in the supernatant was removed by a rotary evaporator. Then, the remaining suspension was preserved for vacuum drying at 50.0 °C until that mass was constant. The 60% Tartary buckwheat flavonoids, namely, CF, was obtained.

### 2.3. Purification by ASR

**Figure 2:**
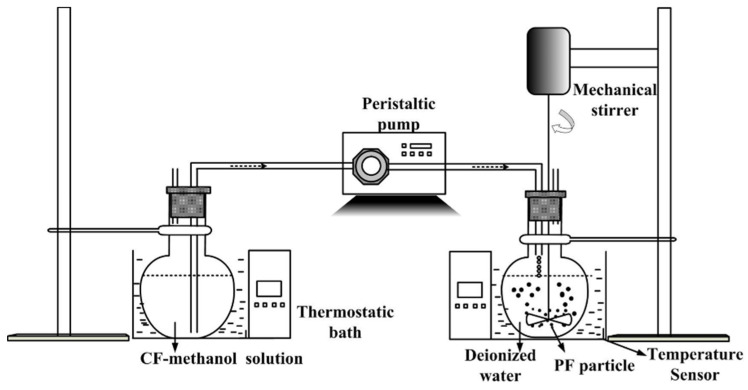
Schematic description of antisolvent operation procedure.

The specific antisolvent equipment is shown in Figure 2. A certain weight of CF as previously stated was weighed and added to methanol by 30.0 mg/mL of TF, and the insoluble residue was filtrated to obtain clear solution. Next, the solution was pumped into deionized water by peristaltic pump under stirring with constant speed. After a while, all solutions were removed by filtration, and the precipitates were obtained and washed thrice using deionized water. The PF was collected after oven drying at 120.0 °C at constant mass.

### 2.4. Box–Behnken Design (BBD)

The CF refinement procedure was optimized by BBD and illustrated in Table 1. The four factors include the stirring time (X_1_), TF concentration (X_2_), recrystallization temperature (X_3_), and the volume ratio of antisolvent to solvent (X_4_). The levels of those independent variables were based on preliminary experiments. The yield (Y_1_) and purity (Y_2_) of TF were considered as response variables. The experimental design was generated and evaluated using Design-Expert^®^ Version 8.05b software (State-Ease Inc., Minneapolis, MN, USA).

The response variables and test variables were related by a second-order polynomial equation for the TF yield, and the contents are displayed as Equations (1) and (2).

(1) $$Y_{1}=82.27+15.00X_{1}+24.88X_{2}-14.48X_{1}X_{2}-10.26X_{1}X_{3}+8.56X_{3}X_{4}\;-11.58X_{2}{}^{2}-8.37X_{3}{}^{2}-6.54X_{4}{}^{2}\;$$

(2) $$Y_{2}=95.89-1.07X_{2}+2.34X_{1}X_{2}+5.43X_{2}X_{4}-1.99X_{2}{}^{2}-1.73X_{4}{}^{2}\;$$

### 2.5. Detection of TF Content

The colorimetric method reported by Yu et al. [29] with slight modification was used to detect TF content. PF was dissolved in methanol to obtain sample solution. A sample measuring 1.0 mL and 5% NaNO_2_ solution were added into a flask, blended, and allowed to sit for 6 min. Subsequently, 1.0 mL 10% Al(NO_3_)_3_ solution was set into the flask, blended, and allowed to leave for 6 min. Finally, 3.0 mL 5% NaOH solution was added, and the volume was adjusted to 10.0 mL with deionized water, blended, and examined after 15 min. The detection wavelength was 500 nm against the same mixture, without the sample as a blank. The amount of the TF was expressed as RU. The calibration curve range is 14.469–463.000 μg/mL.

### 2.6. Identification of the Main Flavonoids

The modified high-performance liquid chromatography (HPLC) method was performed on a Waters chromatographic instrument (Waters Delta 600 pump and a 2487 UV detector) [30]. The chromatographic column is a C_18_ reverse-phase column (250 mm × 4.6 mm, 5 μm, China). The mobile phase omprises 0.1% formic acid in water (A) and 0.1% formic acid in acetonitrile (B) at a flow rate of 0.6 mL/min. The composition of mobile phase was 10–20% (B) for 0–2 min, 20–28% (B) for 2–5 min, 28–50% (B) for 5–8 min, 50–95% (B) for 8–10 min, and 95–10% (B) for 10–12 min. The detection wavelength is 354 nm. The injection volume is 5 μL, and the column temperature is 25 °C. All samples were filtered through 0.22 μm membranes before being injected into an HPLC system.

In order to determine the main flavonoid components, an UPLC system (1290 series, Agilent Technologies, Santa Clara, CA, USA) coupled with an Agilent 6460 Triple Quadrupole Mass Spectrometer (QQQ-MS, Agilent Technologies, Santa Clara, CA, USA) was used to further identify the structure of the main flavonoid in CF and PF. Mass spectrometry conditions were as follows: ion source temperature 100 °C; cone backflush gas flow 50 L/h; capillary voltage 3 kV. The scanning *m*/*z* range was 100–1000, and collision energy was 25 eV. The negative ion mode was chosen for operating the mass spectrometer. The injection volume was 2 μL, and column temperature was set at 35 °C.

### 2.7. Characterization of Morphology

The morphology of CF and PF in optimal experiment condition was examined with SEM (Quanta 200, FEI, Hillsboro, OR, USA). The dry samples were placed on observation desks with a plated-gold conductive layer, and the surface morphology of samples was achieved.

The particle size of PF was performed by a laser particle size analyzer. PF distributed in water was set in glass cuvette for detection. Each measurement was repeated at least thrice for the last 180 s.

### 2.8. Solubility

The solubility (TF solubility) study of CF and PF was performed by colorimetric method. First, excess CF and PF were added into water, simulated gastric fluid (SGF, 0.4% Tween-80 in 1000 mL distilled water; pH = 1.2), and simulated intestinal fluid (SIF, 6.40 g Na_2_HPO_4_·12H_2_O; 0.60 g KH_2_PO_4_; 5.85 g NaCl were mixed in 1000 mL distilled water; pH = 6.8), and then they were placed in a bath pot at 37 ± 0.5 °C for 48 h at a paddle speed of 100 r/min. Second, 2 mL samples were taken out and filtered with 0.22 μm filters for ultraviolet spectrograph detection.

### 2.9. Cellular Antioxidant Activity (CAA) Assay

The HepG2 cells were cultured in Dulbecco’s modified Eagle’s medium (DMEM) with 10% (*v*/*v*) fetal bovine serum (FBS), 100 U/mL penicillin, and 100 mg/mL streptomycin. The cell group was grown at 37 °C in a humidified incubator with 5% CO_2_. After culturing at approximately 48 h intervals, they were applied to the CAA assay.

A cellular ROS experiment was implemented as previously described with some modifications [31]. The cells (5 × 10^4^/well for HepG2) were seeded into a 96-well microplate. After culturing in 5% CO_2_ and 37 °C for 24 h, the medium was removed, and wells were washed with phosphate-buffered saline (PBS). Then, the cells were treated for 1 h at 100 μL of different concentrations of CF or PF dispersion in addition to 25 µM DCFH-DA dissolved in DMEM. Subsequently, the wells were washed twice with 100 μL PBS, and 100 μL oxidant treatment medium containing 500 µM AAPH was added to the cells of the control and experimental groups. Finally, fluorescence microplate readers were used to measure fluorescence intensity at 37 °C every 5 min for 1 h, the emission wavelength was 538 nm and excitation wavelength was 485 nm. CAA was calculated by the following Formula (3):

(3) $$\mathrm{CAA}=\left(1-\frac{\int_{0}^{60}SAdt}{\int_{0}^{60}CAdt}\right)\times 100,$$

where $\int_{0}^{60}SAdt$ is the integrated area under the sample fluorescence versus time curve, and $\int_{0}^{60}CAdt$ is the integrated area under the control group curve [32].

The median effective concentration (EC50) was obtained by calculating from the median effect plot of log (fa/fu), where fa is the fraction affected and fu is the fraction unaffected by the treatment. The EC50 values were stated as mean ±standard deviation for triplicate sets of data obtained from the same experiment.

The HepG2 cells (4 × 10^5^ cells/mL) were inoculated into 6-well plates and cultured for 24 h. The normal and model groups were incubated with 25 µM DCFH-DA and the drug groups were co-incubated with 25 µM DCFH-DA corresponding drugs (CF and PF) for up to 1 h. The treatment was stopped and the wells were washed twice with PBS, then the wells were treated with AAPH of 500 μM for 1 h at 37 °C. Finally, quintic washing was performed with PBS, and the cells were observed by fluorescence microscope [33].

### 2.10. Simulated In Vitro Digestion

A human gastrointestinal digestion model was performed with slight modifications as previously described [34]. The enzymes and digestive juice ingredients required for the digestion model are shown in Table S1. In vitro digestion procedures included the mouth, stomach and small intestine, and E. coli and L. casei were applied to simulate digestion of the large intestine. Te *E. coli* and *L. casei* were cultured Luria–Bertani (LB) Broth and MRS Broth, respectively, at 37 °C for 12 h for activation. After incubation, the final number of bacterial colonies was log 10^8^–10^10^ [29].

The digestion procedures were performed as follows: Ι. 0.2 g samples and 6 mL of simulated saliva solution were mixed in each flask (pH 6.8) and then they were incubated at 37 °C for 5 min. Π. Adding 12 mL of simulated gastric juice (pH 1.5) to the mixture and stirring for 2 h at 37 °C. III. Duodenal juice measuring 12 mL, 6 mL of bile juice, and 2 mL of 70% bicarbonate solution (pH 8.0) were added, and the mixture was stirred for 2 h at 37 °C. IV. Liquid agars measuring 38 mL containing *E. coli* and *L. casei* were used for a sample previously digested in the small intestine step and incubated for 4 h at 37 °C. The digestive samples were shaken using the water bath to simulate the motility of the gastrointestinal tract.

The dissolution rates and residual contents of TF in CF and PF after digesting in the mouth (Ι), gastric (Ι–Π), small intestine (Ι–III), and large intestine digestion (Ι–IV) were determined via HPLC and UV, comparing dissolution and the digestibility of samples during the simulated digestion.

### 2.11. Intestinal Absorption Study

A rat-everted intestinal sac model [35] was used to test the intestinal absorption of CF and PF in vitro. Female Sprague–Dawley rats (approximately 220 g) fasted for 24 h were used throughout the investigation. The abdomen was opened after the rat was anesthetized with intraperitoneal administration of urethane (1000 mg/kg). Then, a 10 cm jejunum segment was obtained (i.e., 2 cm distal to the ligament of Treitz). The segment was flushed with cold and oxygenated saline for cleaning, and an intestinal segment was carefully everted over using a glass rod. One end of the segment was tied with a silk thread, thereby forming a sac. Then, the empty sac was filled with 1 mL of blank Krebs–Ringer’s buffer with pH = 7.3 (KRB, containing 6.90 g NaCl, 0.35 g KCl, 0.29 g MgSO_4_·7H_2_O, 0.16 g KH_2_PO_4_, 2.10 g NaHCO_3_, 0.28 g CaCl_2_, and 0.2% glucose in 1 L of distilled water) and placed into a 20 mL KRB solution containing 333.0 μg/mL CF and 200.0 μg/mL PF (containing 199.8 μg/mL TF). The solution was maintained at 37 °C using bath water. At the incubations of 10, 15, 20, 30, 45, 60, 75, 90, 105, and 120 min, 100 μL of the KRB in empty sac was withdrawn for TF detection by UVS, as described in Section 2.5, and the same volume of blank KRB was supplemented. The experiments were repeated thrice, and absorption rate constant (K_a_, μg·min^−1^·cm^−2^) was calculated by using Equations (4) and (5):

(4) $$Q_{n}={\;C}_{n}+0.1\overset{n-1}{\underset{i=1}{\sum }}C_{i},$$

(5) $$K_{a}=L/A,\;$$

where $Q_{n}$ is the cumulative absorption of the drug at each time (μg); Cn is the actual detection concentration at time point (μg/mL); L is a slope of regression analysis according to the cumulative uptake of the drug and time (μg/min), and A is the absorption surface area (cm^2^) of the intestinal sac.

### 2.12. DPPH Free Radical Scavenging Activity

The DPPH method was applied to evaluate antioxidant activity of CF and PF during in vitro human digestion. Supernatant measuring 2 mL was taken from each step during the simulated digestion and mixed with a 1 mL DPPH solution (0.3 mM). The reaction mixture was incubated for 30 min, and the absorbance was measured at 517 nm. The experiment was repeated 3 times. DPPH scavenging used the following Equation (6).

(6) $$\mathrm{SC}\;\left(\%\right)=\frac{\left(\mathrm{Ac}-\mathrm{Ai}\right)}{\mathrm{Ac}}\times 100\%\;$$

Here, A_c_ and A_i_ are the absorbance of the control and the samples, respectively.

### 2.13. Detection of Lipid Oxidation

All experiments were completed while complying with the relevant laws and institutional guidelines. The four-month-old Balb/C mice with average weights of approximately 50 g were raised for a week to adapt in standard fed conditions; the mice sacrificed by CO_2_ gas and the liver were excised and rinsed with chilled 0.2 M phosphate buffer (pH 7.2). The liver (0.1 g) was homogenized in 0.2 M phosphate buffer saline (pH 7.2) in an ice bath, and the homogenate was centrifuged at 10,000× *g* for 10 min at 4 °C. Aliquots (0.1 mL) of the supernatant were incubated with the test samples (0.2 mL) in the presence of 10 μM ferrous sulfate (0.1 mL) and 0.1 mM vitamin C (0.1 mL) at 37 °C for 1 h. The MDA content in the supernatant was quantified with the thiobarbituric acid method by a lipid peroxidation MDA assay kit. The samples were measured at 532 nm. The inhibition ratio (%) was analyzed via follow Equation (7):

(7) $$\mathrm{IR}\;\left(\%\right)=\frac{\left(\mathrm{Ac}-\mathrm{Ai}\right)}{\mathrm{Ac}}\times 100\%\;$$

Here, A_c_ and A_i_ present the absorbance of the control and the samples, respectively.

## 3. Results

### 3.1. Analysis of BBD Models

**Figure 3:**
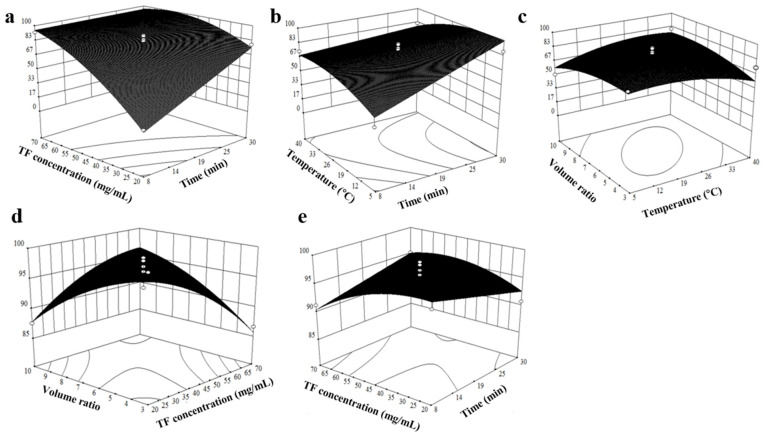
RSM plots—(**a**) Effect of TF concentration and stirring time; (**b**) effect of temperature and stirring time; (**c**) effect of temperature and volume ratio of water and methanol on PF yield; (**d**) effect of total flavonoids and volume ratio of water and methanol; (**e**) effect of TF concentration and stirring time on PF purity.

On the basis of the BBD matrix listed by Design-Expert software, 30 experiments were carried out to estimate the sum of squares of pure errors (Table 2 and Table 3). The “Lack of fit” *p* value > 0.05 of (0.1566, 0.3442) indicates that the lack of fit is not significantly related to the pure error, which shows that the quadratic regression model fits well with actual situation. Response surface plots in contour and three-dimensional plots were used to identify the relationship between factors and responses (Figure 3a–e). For optimizing the input factors, Derringer’s desirability function method was applied. The desirability function identifies a combination of the variable levels that optimizes a set of responses. The desirability (d) for each experiment is carried out by declaring the targets and boundaries important for each answer. The process directed that the stirring time of 13.9 min, the concentration of TF of 70.0 mg/mL, the recrystallization temperature of 38.0 °C, and the volume ratio of antisolvent to solvent of 10:1 will yield the desired result, and purities of TF were 94.34% and 99.82% individually with an overall desirability value of 0.998. To verify the accuracy and fitness of models, the optimal process of purity was repeated thrice. The mean value of three outcomes is as follows: the yield of PF was 94.35%, and the purity of PF was 99.81%. Thus, the experimental value was consistent with the anticipated value of optimal models. The stirring time was 14 min. Thus, the experimental operation was precise in the final confirmation. All follow-up tests were performed on PF obtained under the above-mentioned conditions.

### 3.2. Content TF and Main Flavonoid Compounds

**Figure 4:**
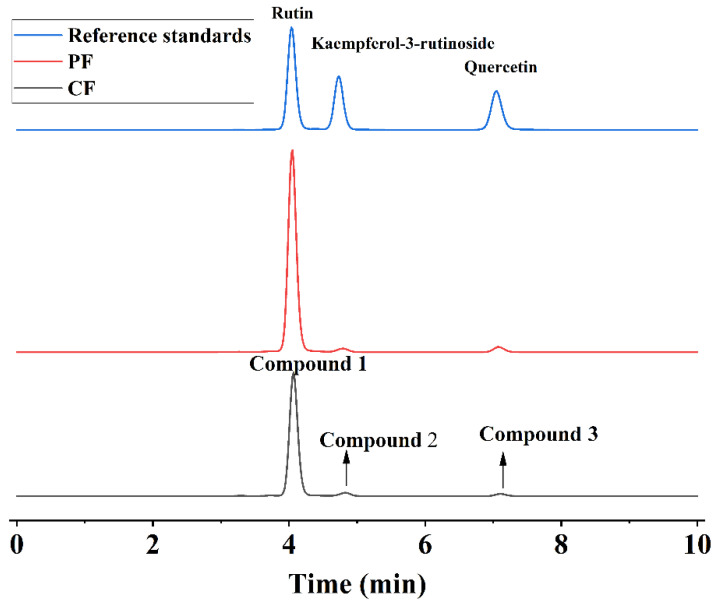
HPLC chromatogram: reference standards of three flavonoids (blue line), the main flavonoids in PF (red line), and CF (black line).

**Figure 5:**
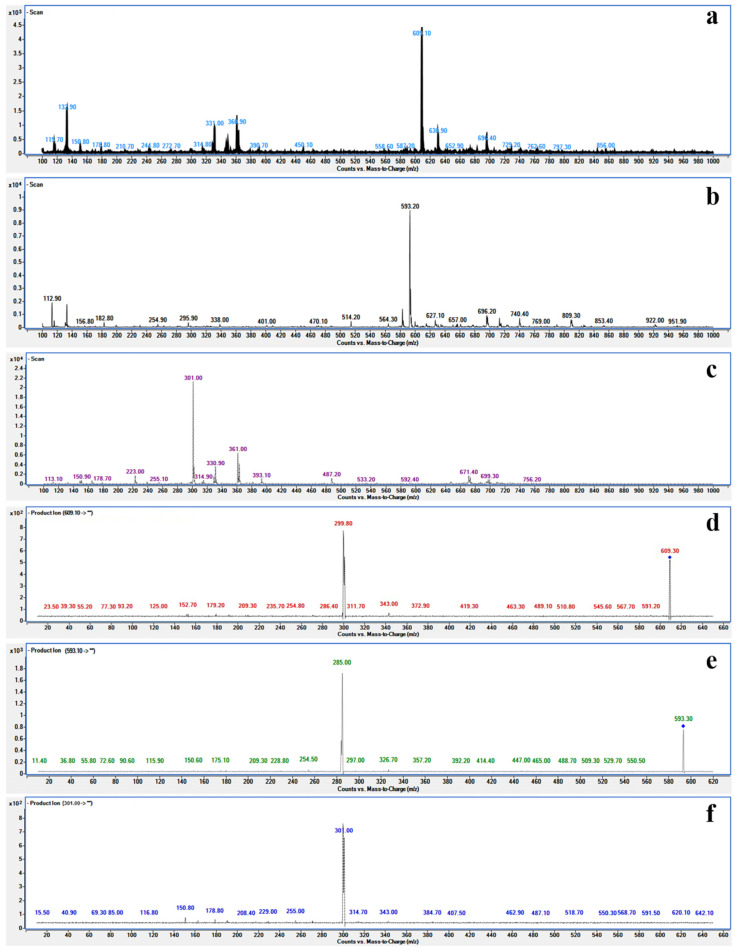
The UPLC-MS/MS chromatograms of three compounds (**a**–**c**); MS/MS fragmentation of three compounds (**d**–**f**) in negative ion mode.

There are three peaks of the compound in the results of HPLC for CF and PF (Figure 4). After mass spectrometry analysis, their precursor and product ions are shown in Figure 5 and Table 4, the *m*/*z*[M-H]^−^ for each was 609.01 (1), 593.20 (2), and 301.00 (3). Combining MS/MS data and previous literature [36,37], we speculated these substances to be RU, KA, and QU, respectively. Finally, we confirmed that they are the main flavonoids of CF by comparison with the HPLC result (Figure 4) of the standard. The suitability tests of the HPLC method including reproducibility, linearity, detection limit, precision, stability, and recovery have been carried out before the extracts were determined. The limits of detection of RU, QU, and KA were 0.65, 2.60, and 1.20 μg/mL, respectively. The recoveries for the three flavonoids were between 98.26% and 99.98%. The linear regression equations of RU, QU, and KA were y = 10981.5409x − 43.7104 (R^2^ = 0.9999, 20–100 μg/mL), y = 15940.4056x − 47.5222 (R^2^ = 0.9998, 10–50 μg/mL), and y = 10057.5529x − 19.7159 (R^2^ = 0.9998, 20–100 μg/mL), respectively.

The content of PF under the optimal condition was detected by UV spectrophotometry, which was raised significantly to 99.81% from 60%. In addition, RU, QU, and KA increased at approximately 1.63 times, 1.42 times, and 1.60 times in PF, respectively (Table 5). The reason that these issues were improved is that the TF of CF can be dissolved in methanol first. In addition, some insoluble substances have been separated from CF, such as polysaccharide. In addition, other impurities in methanol solution cannot be recrystallized when the solvent was added into deionized water. Compared with other studies and developments, the PF yield in this study in purified CF was also enhanced obviously, whereas that of flavonoids increased by applying ASR.

### 3.3. Morphology Observation and Particle Size Analysis

**Figure 6:**
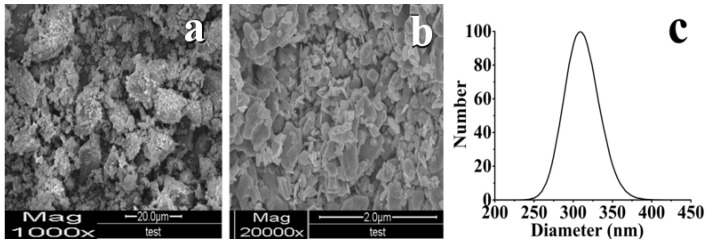
SEM photos and particle size distribution of sample morphology: (**a**) CF; (**b**) PF; (**c**) particle size distribution.

The SEM photos of sample morphology are exhibited in Figure 6. In Figure 6a, CF showed irregular block particles at a diameter of approximately 1250–2470 nm. From Figure 6b, PF morphology showed minimal sphere-like and strip shapes with particle sizes of approximately 250–430 nm, which is a more uniform distribution. This result indicated that the PF obtained by antisolvent reduced in size compared with CF because TF recrystallization was slow after nucleation in water–methanol systems. From Figure 6c, the particle size distribution demonstrated a mean diameter of 309.7 nm, which adapts SEM result. The decrease in particle size may improve the solubility of TF [32,38].

### 3.4. Solubility Analysis

**Figure 7:**
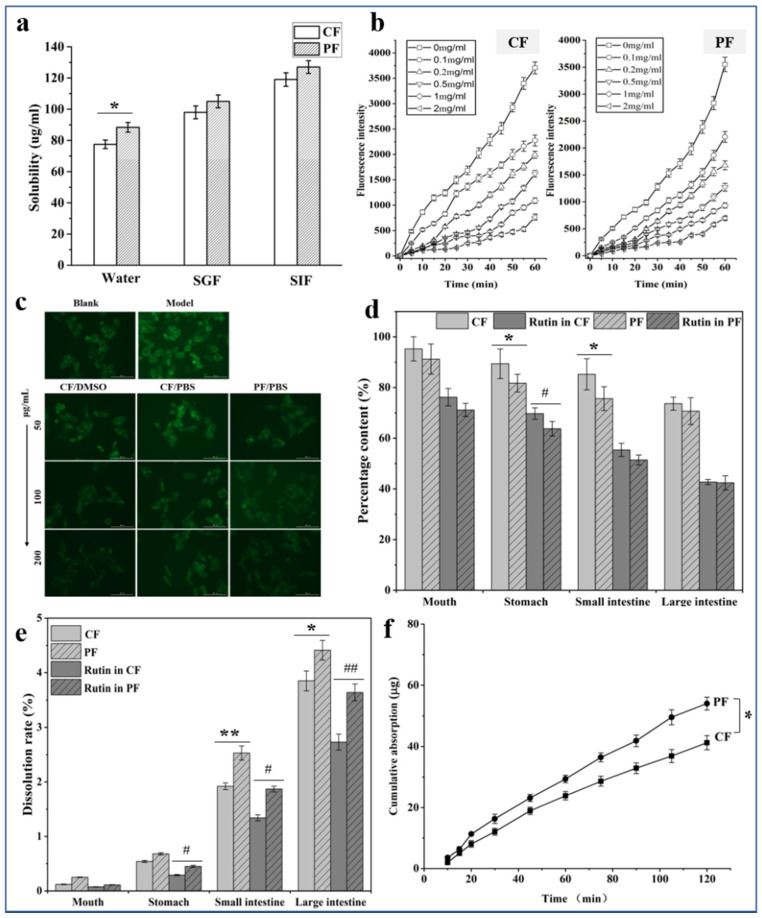
(**a**) Equilibrium solubility of total flavonoids of CF and PF in water, SGF, and SIF (means ± SD, n = 3, * *p* < 0.05 compared with CF). (**b**) Fluorescence intensity after treatment at different concentrations of CF and PF over time. (**c**) The images of HepG2 cells incubated with CF or PF for 50, 100, and 200 μg/mL, which were measured by fluorescence microscope. (**d**) The residual amount of CF and PF after digestion, and the residual amount of RU in CF with PF after digestion. (means ± SD, n = 3, * *p* < 0.05 compared with CF, ^#^ *p* < 0.05 compared with rutin in CF). (**e**) Dissolution rate of CF and PF after digestion in vitro, and dissolution rate of CF with PF after digestion in vitro (means ± SD, n = 3, * *p* < 0.05, ** *p* < 0.01 compared with CF, ^#^ *p* < 0.05, ^##^ *p* < 0.01 compared with rutin in CF). (**f**) Cumulative absorption profiles of CF and PF in the intestinal sac model. (means ± SD, n = 3, * *p* < 0.05 compared with CF).

The solubility (solubility of flavonoids) of CF and PF tested by UVS in three media is illustrated in Figure 7a. The equilibrium solubilities of CF and PF were 77.5 and 88.4 μg/mL in the water. The SGF and SIF solubilities of CF were 98.0 and 105.0 μg/mL, whereas those of PF were 119.0 and 127.0 μg/mL, respectively. Thus, PF solubility in the three media all increased compared with that of CF, which was attributed to the reduction in particle diameter enabling PF to have great chance to contact the molecules of the three media.

### 3.5. Cellular Antioxidant Analysis

From Figure 7b, the fluorescence intensity became weak with the increasing doses of samples. This phenomenon showed that flavonoids from Tartary buckwheat possess antioxidant activity. In addition, EC50s of CF and PF were calculated when CAA = 50, which were 172 and 140 μg/mL, respectively. Micronized drugs show large surface areas for attachment to the solution medium, causing high solubility at the same time period. Thus, PF shows a superior antioxidant activity. This finding is valuable for antioxidant development.

Fluorescence photographs of the HepG2 cells (Figure 7c) showed that the normal control group had weak fluorescence, whereas the model group had strong fluorescence, which indicated that ROS was generated in the presence of AAPH in the cells. However, the intervention of the drugs (CF and PF) resulted in a dramatic reduction in ROS. Simultaneously, we observed that oxidative stress damage is negatively correlated with the concentration of flavonoids. When the flavonoids’ concentration increased from 50 to 200 μg/mL, intracellular fluorescence intensity gradually weakened in the drugs’ group. Compared with the CF/PBS group, the fluorescence intensity in the cells of the PF/PBS group was relatively lower at the same concentration as a result of higher solubility of micronized flavonoids. The fluorescence intensity displayed in CF/DMSO group cells confirmed this conjecture. It is worth mentioning that the smaller particle size of drug, the easier cellular uptake is, making it more capable of cellular internalization. Taken together, these data demonstrated that the micronized PF obtained by ASR increases ROS scavenging activity.

### 3.6. Analysis of Simulated In Vitro Digestion

After four simulated digestion steps, the remaining amount of TF content was shown in Figure 7d. The TF components for CF were 95.25%, 89.37%, 85.21%, and 73.62% in mouth, stomach, small intestine, and large intestine, respectively, and those for PF were 91.22%, 81.72%, 75.63%, and 70.68% in the above four corresponding environments. The PF was digested faster than CF in all steps due to the higher solubility in water, resulting in lower TF content in the digestion solution. Another obvious result was shown in Figure 7d, and RU was largely destroyed in TF, while the content of TF was not severely affected, indicating that RU could have a structural transformation after being digested. Flavonoids are highly sensitive to pH changes of the digestive juice, particularly relative to the mild alkaline conditions in the small intestine. Therefore, during digestion in the duodenum, a portion of these compounds may be transformed into different structural forms that have different chemical properties [39].

In addition to understanding the effect of particle size of TF on digestibility, we tested the dissolution rate of TF at each digestion step via simulating the digestion mode in vitro. It can be found that the TF showed good dissolution rates in small and large intestines, 1.92% and 3.85% for CF and 2.53% and 4.41% for PF, which further confirmed the reason for the higher digestibility of TF in both intestinal fluids. Meanwhile, as the main compound of flavonoids, RU has the same rule of dissolution with TF. From the above results, PF has a smaller particle size than CF, for which its solubility and dissolution rate has been improved because of the larger surface area. In particular, in alkaline conditions, the effect of particle size on digestibility is more obvious.

### 3.7. In Vitro Intestinal Absorption

The pretest (not be shown) was examined to ensure the viability of the intestinal sacs and experiment accuracy. The result showed that the tissue of intestinal sac model was viable during periods of ex vivo permeability assays. Notably, this method can be well applied to the ex vivo-everted rat gut sac permeability assay to evaluate the oral transport (permeability) characteristics of the two flavonoid samples. Figure 7f shows the linear increase in cumulative absorption of the two samples at the incubation time of up to 120 min. However, PF has a higher K_a_ (0.27 μg·min^−1^·cm^−2^) than CF’s (0.21 μg·min^−1^·cm^−2^). The cause of such rapid absorptions of PF can be the fast dissolution rate. Thus, this result also indicated that micronized PF can be well absorbed in vivo due to decreased particle size.

### 3.8. Effect on Antioxidant Activity of TF Simulated In Vitro Digestion

**Figure 8:**
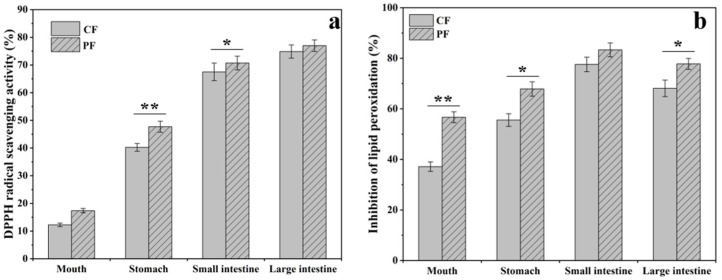
DPPH (**a**) and MDA (**b**) of CF and PF as they pass through an in vitro human digestion model (means ± SD, n = 3, * *p* < 0.05, ** *p* < 0.01 compared with CF).

The result of DPPH free radical scavenging is presented in Figure 8a. After in vitro human digestion in the stomach and intestines, the antioxidant activity values of TF increased dramatically, the scavenging rates of DPPH free radicals by CF were from 12.33% to 74.86%, and PF were from 17.36% to 76.97%, respectively. However, antioxidant activity was not influenced by digestion in the mouth. This may be due to the hydrolysis of flavonoids glycosides to produce the aglycone under acidic conditions, and the antioxidant activity of aglycone is better than that of flavonoids glycosides [40,41]. Next, the effect of digestive enzymes and enterobacteria promotes the further decomposition of flavonoids glycosides into aglycone, and the solubility of aglycone increased under alkaline conditions, resulting in an improvement in the antioxidant activity of flavonoids [42].

The inhibitory effect of TF on lipid oxidation in mouse brain lipids is presented in Figure 8b. The inhibition of lipid oxidation of TF increased after digestion in the stomach for both CF and PF. This was attributed to the increase in the amount of aglycone in the TF during in vitro human digestion, and the penetration of aglycone in the cell membrane was better than flavonoids glycosides. In addition, it can be seen from the above results that the antioxidant activity of digested PF is still higher than that of CF due to the increase in solubility. Moreover, micronized flavonoids also provide additional pathways for cellular uptake to make more PF to be captured by cells.

## 4. Discussion

ASR technology was used to treat CF from TBS; finally, the purity of TF was improved. Its purity and yield are related to recrystallization temperature and time, solvent and anti-solvent volume ratio, and total flavone concentration. The mechanism of this technology mainly lies in the different supersaturation of substances in the solvent system, resulting in different crystallization orders to separate and purify the target product. In previous research, the Tartary buckwheat bran and Tartary buckwheat flour were treated by the traditional ultra-fine powder process (shear crushing, air flow crushing, and wet grinding) to obtain the powder with uniform small particle size. Due to a larger contact area with the solution, the hydration performance and antioxidant activity of micronized powder were increased [43,44,45]. In this study, the ASR technology can reduce particle size and increase the specific surface area by controlling the nucleation rate of TF. In the process of digestion, the interaction between micronized TF and digestive enzymes was enhanced, which can accelerate the formation of aglycones, promote the improvement of antioxidation ability, and improve human health.

## 5. Conclusions

In the present study, micronized flavonoids of high purity from Tartary buckwheat seeds were successfully achieved by the ASR method. BBD was applied to obtain optimal purification conditions: stirring time (X_1_) of 13.9 min, concentration of TF (X_2_) of 70.0 mg/mL, recrystallization temperature (X_3_) of 38.0 °C, and volume ratio of antisolvent to solvent (X_4_) of 10:1. We found that the main flavonoid composition of PF was almost the same as that of CF, but the content of TF increased from 60.00% to 99.81%, and the yield was 94.35%. Compared with CF, PF has higher solubility and antioxidant activity in vitro due to the reduction in the micronized particle size of TF. In addition, the better solubility promotes the dissolution rate and digestibility of PF in vitro human digestion model. After being digested by the gastrointestinal tract, CF and PF still have high antioxidant activities, and it is important that the antioxidant capacity of PF after digestion is even better. These results concluded that ASR may be an effective method for purifying and micronizing natural products.

## Figures and Tables

**Table 1:** The factors and levels of in response surface methodology.

Factor	X_1_	X_2_	X_3_	X_4_
	Stirring Time (min)	Total Flavonoids Concentration (mg/mL)	Recrystallization Temperature (°C)	Volume Ratio of Antisolvent to Solvent (*v*:*v*)
Code levels				
−1	8.0	20.0	5.0	3.0
0	19.0	45.0	22.5	6.5
1	30.0	70.0	40.0	10.0

**Table 2:** Analysis of variance of TF yield model.

Source	Sum of Square	Degree of Freedom	Mean Square	F-Value	*p*-Value	Significance
Model ^a^	13,087.32	8	1635.92	22.26	<0.0001	significant
X_1_	2700.3	1	2700.3	36.75	<0.0001	
X_2_	7426.18	1	7426.18	101.06	<0.0001	
X_1_ X_2_	838.39	1	838.39	11.41	0.0028	
X_1_ X_3_	421.07	1	421.07	5.73	0.0261	
X_3_ X_4_	293.09	1	293.09	3.99	0.0589	
X_2_^2^	938.59	1	938.59	12.77	0.0018	
X_3_^2^	489.9	1	489.9	6.67	0.0174	
X_4_^2^	291.62	1	291.62	3.97	0.0595	
Residual	1543.21	21	73.49			
Lack of Fit	1372.69	16	85.79	2.52	0.1566	Not significant

The results were obtainedwith Design Expert 8.05b software. ^a^ X_1_: stirring time; X_2_: TF concentration (mg/mL); X_3_: recrystallization temperature (°C); X_4_: methanol–water ratio (*v*/*v*); Y_1_: TF yield (%) Y_2_: TF yield content (%).

**Table 3:** TF content model variance analysis.

Source	Sum of Square	Degree of Freedom	Mean Square	F Value	*p*-Value	Significance
Model ^a^	198.26	5	39.65	8.59	<0.0001	significant
X_2_	13.85	1	13.85	3	0.0961	
X_1_X_2_	21.81	1	21.81	4.72	0.0398	
X_2_X_4_	118.05	1	118.05	25.57	<0.0001	
X_2_^2^	28.2	1	28.2	6.11	0.0209	
X_4_^2^	21.25	1	21.25	4.6	0.0422	
Residual	110.79	24	4.62			
Lack of Fit	94.33	19	4.96	1.51	0.3442	not significant

The results were obtained with Design Expert 8.05b software. ^a^ X_1_: stirring time; X_2_: TF concentration (mg/mL); X_3_: recrystallization temperature (°C); X_4_: methanol–water ratio (*v*/*v*); Y_1_: TF yield (%) Y_2_: TF yield content (%).

**Table 4:** Chemicals in CF identified by UPLC-MS/M.

Compound	Polarity	Precursor (*m*/*z*)	Product (*m/z*)
1	Negative	609.10	299.80
2	Negative	593.20	285.00
3	Negative	301.00	150.80

**Table 5:** The content of main flavonoids in CF and PF by HPLC.

Samples	RU (%)	QU (%)	KA (%)
CF	52.78	3.67	2.26
PF	86.37	5.21	3.61

## Data Availability

The data presented in this study are available in this article and Supplementary Material.

## Supplementary Materials

The following are available online at https://www.mdpi.com/article/10.3390/foods11091346/s1, Table S1: Constituents and concentrations of the various simulated juices used in in vitro human digestion model.
