# Supplementary material for: Antioxidative Activity Evaluation of High Purity and Micronized Tartary Buckwheat Flavonoids Prepared by Antisolvent Recrystallization
Source: Foods. 2022 May 5;11(9):1346. doi: 10.3390/foods11091346 (PMC9102898; doi:10.3390/foods11091346)
Supplement: Supplementary file 1 [file foods-11-01346-s001.zip › foods-1681692-supplementary.pdf]

Antioxidative activity evaluation of high purity and micronized tartary buckwheat flavonoids prepared by antisolvent recrystallization

Yanjie Liu [1,2,3], Xiaoyu Sui[4*], Xiuhua Zhao [1,2,3,*], Siying Wang [1,2,3] and Qilei Yang [1,2,3]

[1] College of Chemistry, Chemical Engineering and Resource Utilization, Northeast Forestry University, Harbin 150040, China; klp15lyj@nefu.edu.cn (Y.L.); wsy0822@nefu.edu.cn (S.W.); yql@nefu.edu.cn (Q.Y.); zhangxiaoxue@nefu.edu.cn (X.Z.)
[2] Key Laboratory of Forest Plant Ecology, Ministry of Education, Northeast Forestry University, Harbin 150040, China
[3] Heilongjiang Provincial Key Laboratory of ecological utilization of Forestry-based active substances, Northeast Forestry University, Harbin 150040, China
[4] College of Pharmacy, Qiqihar Medical University, Qiqihar 161006, China
* Correspondence: suixiaoyu@outlook.com, xiuhuazhao@nefu.edu.cn

Table S1. Constituents and concentrations of the various simulated juices used in in vitro human digestion model.

	Saliva (Step I)	Gastric juice (Step Π)	Duodenal juice (Step Ш)	Bile juice (Step IV)
**Organic and inorganic components**	1.7 mL NaCl (175.3 g/L)	6.5 mL HCl (37 g/L)	6.3 mL KCl (89.6 g/L)	68.3 mL $NaHCO_3$ (84.7 g/L)
	8 mL urea (25 g/L)	18 mL $CaCl_2$ $2H_2O$ (22.2 g/L)	9 mL $CaCl_2$ $2H_2O$ (22.2 g/L)	10 mL $CaCl_2$ $2H_2O$ (22.2 g/L)
	15 mg uric acid	1 g bovine serum albumin	1 g bovine serum albumin	1.8 g bovine serum albumin
				30 g bile
**Enzymes**	290 mg α-amylase	2.5 g pepsin	9 g pancreatin	
	25 mg mucin	3 g mucin	1.5 g lipase	
**pH**	6.8 ± 0.2	1.50 ± 0.02	8.0 ± 0.2	7.0 ± 0.2

After mixing all ingredients, the volume was augmented to 500 mL with distilled water. If necessary, the pH of the juices was adjusted to the appropriate value.
